# Quantitative ethnobotany of plants used for vernacular building construction in Ghana

**DOI:** 10.1371/journal.pone.0313778

**Published:** 2024-11-15

**Authors:** Maxwell Kwame Boakye

**Affiliations:** Department of Environmental Science, Ho Technical University, Ho, Ghana; Kwame Nkrumah University of Science and Technology Faculty of Pharmacy and Pharmaceutical Sciences, GHANA

## Abstract

Vernacular construction uses locally available materials, including plant-based materials. The sustainability of plant use raises concerns. Social evaluation using an ethnobotanical approach gives a clear overview of the sustainability of wild plant harvesting as it incorporates the views of resource users. In Ghana, plant-based materials are commonly used for vernacular housing construction, especially in rural areas. However, the cultural significance of plant species used for vernacular building construction remains unexplored. This study aimed to take an inventory of plant species and use their ethnobotanical indices to evaluate the sustainability of utilization in vernacular building construction. Ethnobotanical data on plant species used for vernacular building construction was collected from 258 respondents in the Adaklu district of the Volta region. Quantitative ethnobotanical analysis revealed that of the 26 plant species identified to be used for vernacular building construction, *Bambusa vulgaris*, *Borassus aethiopum*, *Elaeis guineensis*, *Senna siamea*, and *Azadirachta indica* were the most culturally significant and valuable. *Bambusa vulgaris* had the highest cultural importance, value, and relative importance index. *Elaeis guineensis* had the highest relative frequency of citations. The findings reveal a high potential for valorizing the culturally important and valuable plant species in the local construction industry. Promoting culturally significant and valuable plant species commercialization presents an opportunity for poverty alleviation at the local level, as most of the species are abundant in the natural environment and are among the commonly planted species for the afforestation program in Ghana.

## Introduction

Vernacular construction provides the needed shelter with traditional construction techniques for people to build their houses using locally available materials [1–4]. A vernacular architecture consisting of mud houses, wattle and daub, and mud-block construction with a thatched roof remains the most common housing type throughout rural Africa [5, 6]. Natural resource exploitation is a major source of material, fuel, and medical supplies for rural African communities with limited access to modern services and goods [7, 8]. The reliance on natural resources for materials, including those used for building, facilitates the high prevalence of vernacular architecture.

Plant-based building materials constitute a prominent and indispensable component of a vernacular building [1–4, 9]. The dependence on plant-based materials for vernacular construction raises the issue of the sustainability of their extraction and utilization for building materials. The availability of species, their regeneration potential, and the rate of use of the species have been the scientific focus of sustainable utilization of plants in building construction [4, 9, 10]. However, most resources are used by local people, which, in turn, requires an understanding of their perspective on resource sustainability. Social evaluation using an ethnobotanical approach gives a clear overview of the sustainability of wild plant harvesting from a social evaluation perspective [3, 4, 11].

The ethnobotanical approach identifies and evaluates the plant species that are most important to a given local community’s culture [12–15]. The relative ethnobotanical importance of plants is pertinent to conservation biology as it may indicate the species that may be subjected to the most significant harvesting pressure [12, 14, 16]. Identifying the relative importance of plant species for vernacular building construction may be vital to the social evaluation of sustainability. Quantitative ethnobotanical analysis can assist in identifying plant species under pressure from cultural utilization and requiring mitigation measures. Knowledge of the most culturally valuable plant species can serve as a basis for promoting sustainable utilization and initiating conservation management measures for species under pressure in local communities.

In Ghana, plant-based materials are exploited for vernacular building purposes [1]. However, studies focusing on the ethnobotanical description and their quantitative approach to the plant species used for vernacular building construction valuable to the local communities are lacking. Raw material consumption for construction purposes is one of the most severe impacts of construction-related activities on the environment in Ghana [17, 18]. Information on the preferences of plant species for vernacular building purposes among local communities is vital to knowing about the sustainability of utilization and devising a conservation management strategy. This study aimed to take inventory of the plant species used for vernacular buildings and determine their ethnobotanical importance in the Adaklu district of Ghana. The study objectives are to determine the (a) cultural importance and value of the plant species used to construct vernacular buildings, (b) consensus on the use of plant species in building elements, and (c) conservation implications of plant species used in vernacular building construction. Knowledge about the culturally essential plant species is necessary to develop management strategies to ensure the long-term sustainability of the plant species used.

## Materials and methods

### Study area

The study was undertaken in the Adaklu District of the Volta Region of Ghana, which is located at longitudes 06°41′1″N and 6.68361°N and latitudes 00°20′1″E and 0.33361°E. The study area shares boundaries with Ho Municipal to the North, Central Tongu District to the South, Agotime-Ziope District to the East and the West with Ho West District [19]. There are 38,649 people living in the Adaklu District, which is entirely rural, lacks an urban area, and covers a total land area of 810 km^2^ [20]. Most of its residents depend on the natural resources in the area for their livelihood and employment. Extracting poles, beams, and laths for constructing vernacular buildings is a common form of natural resource utilization. Among the materials used to construct the outer walls in the district, wood ranks third, while palm leaf and raffia ranked second and bamboo third as the primary materials used for roofing [19]. The prevalent use of plant-based material for vernacular building construction is anticipated to result in high knowledge of valuable species.

### Ethnobotanical survey

A purposeful and snowball sampling approaches were used to select participants in this study. Participants were chosen based on their familiarity with the study’s topic and their ability to recommend other members of the targeted population [21]. All participants selected were to have been involved in vernacular building construction before or currently. These approaches ensured that the chosen participants were representative of artisans in the vernacular building sector and could provide information pertinent to this study. Based on these two sampling approaches, 311 participants were identified to be actively involved or have been active participants in vernacular building constructions and, therefore, potential candidates for this study. Of these, 258 consented to take part in the study after being informed of their freedom to decline participation at will through an informed oral consent procedure.

Ethnobotanical data on plant-based materials used for vernacular building construction were collected through semi-structured interviews in April and May 2023. The questionnaire focused on the plant species used to construct the main elements of a vernacular building in Ghana, i.e., main poles, main beams, roof laths, wall laths, binding material, and thatching material. The respondents were asked to provide a list of the plant species used for the main beams, which form the primary support structure of the roof; main posts, which support the walls and roof; wall laths, which are sticks woven between two main posts to form the wall structure and may be plastered; binding/tying material, which is used to fasten wall and roof laths; and thatching material, which is used for roofing. The respondents were encouraged and prompted for information using prompts and probing. The interviews were conducted in Ewe, the most widely spoken language in the district, and adopted as a lingua franca for many respondents who could not speak the English language. All the research assistants were fluent in the English and Ewe languages.

The local names mentioned by the respondents were matched with those in the available literature for plant species identification [22–24] and plants identified in the local area [25]. Plant material was collected and used to confirm their identification by comparison with voucher specimens by the curators at the Institute of Traditional and Alternative Medicine, University of Health and Allied Sciences (UHAS). Plants of the World Online (https://powo.science.kew.org) electronic databases was used to establish the names and authority of plant species.

#### Ethics statement

This study was approved by the Ho Technical University ethics committee (Reference HTU/EC 2023–024). The participants gave their oral informed consent before the interview.

#### Data analysis

The analysis of ethnobotanical data was carried out using the "ethnobotany" R package developed by Whitney [26]. Several indices were determined to assess the ethnobotanical significance of different plant species, including the Use Report (UR), Cultural Importance (CI), Frequency of Citation (FC), Number of Uses (NU), Relative Frequency of Citation (RFC), Relative Importance Index (RI), Cultural Value of Ethnospecies (CVe), and Fidelity Level (FL). UR values for each plant species were calculated by counting the number of informants who mentioned each use category for the species and summing up all the uses in each category [27]. The Cultural Importance (CI) index was used to calculate the cultural significance of each plant species in the dataset [12]. The Number of Uses (NU) per plant species was calculated by adding up all the different uses of a species, while the Frequency of Citation (FC) per species was calculated by summing up the number of informants who cited a use for that plant species in the dataset [27]. The Relative Frequency of Citation (RFC) index determined the importance of each plant species based on the number of informants who reported using it, while the Relative Importance (RI) index calculated the relative importance of each species in the dataset, taking into account only the use categories [12]. A species’ cultural, practical, and economic importance were determined using the Cultural Value (CVe) index [15]. Finally, the Fidelity Level (FL) index calculated the percentage of informants who used a plant for the same purpose compared to all the uses of that plant [28]. All these indices were used to assess the ethnobotanical significance of different plant species used for vernacular building. The data file used for the analysis is available in the S1 Table.

#### Informant agreement ratio

The informant agreement ratio (IAR) was used to measure the consensus level among the vernacular building artisans for plant species used for a specific building element. The original formula proposed by Trotter and Logan [29] was interpreted as follows:

$$IAR=\frac{Nur-Ns}{Nur-1}$$ (1)

where Nur = is the total number of use reports (UR) recorded for a given building element, and Ns = is the total number of plant species utilized for the particular building element. The IAR varies between 0 (where there is no consensus or agreement on the number of plant species used for the building element) and 1 (there is consensus on the number of plant species used for the building element).

## Results

A total of 26 plant species were documented to be used for the various construction elements of vernacular buildings (Table 1).

**Table 1:** Ethnobotanical indices of plants used for vernacular building in Ghana.

Family	Scientific name	Local name	Voucher ID	Conservation status	URs	CI	FCs	NUs	RFCs	RIs	CVe
Poaceae	*Bambusa vulgaris Schrad*. *ex J*.*C*.*Wendl*.	Pamploti	UHAS/ITAM/2023/L029	LC	440	1.705	162	4	0.628	0.823	0.714
Arecaceae	*Borassus aethiopum* Mart.	Agorti	UHAS/ITAM/2023/L030	LC	406	1.574	211	2	0.818	0.670	0.429
Arecaceae	*Elaeis guineensis* Jacq.	Deti	UHAS/ITAM/2023/L001	LC	397	1.539	251	2	0.973	0.750	0.499
Fabaceae	*Senna siamea* (Lam.) H.S.Irwin & Barneby	Zangarati	UHAS/ITAM/2023/L028	LC	288	1.116	107	3	0.415	0.588	0.231
Meliaceae	*Azadirachta indica* A.Juss.	Liliti	UHAS/ITAM/2023/L003	LC	265	1.027	103	3	0.399	0.580	0.205
Apocynaceae	*Funtumia africana* (Benth.) Stapf	Kpomi	UHAS/ITAM/2024/SB001	LC	199	0.771	138	2	0.535	0.525	0.138
Cactaceae	*Rhipsalis baccifera* (J.S.Muell) Stearn	Adzorka	UHAS/ITAM/2024/L001	LC	187	0.725	187	1	0.725	0.498	0.088
Arecaceae	*Raphia palma-pinus* (Gaertn.) Hutch.	Ebe	UHAS/ITAM/2024/L002	NT	175	0.678	175	1	0.678	0.474	0.077
Moraceae	*Antiaris toxicaria* (J.F.Gmel.) Lesch.	Logo	UHAS/ITAM/2024/SB002	LC	163	0.632	163	1	0.632	0.450	0.067
Phyllanthaceae	*Bridelia ferruginea* Benth.	Akamiti	UHAS/ITAM/2023/SB001	LC	137	0.531	137	1	0.531	0.398	0.047
Fabaceae	*Baphia nitida* G.Lodd.	Toti	UHAS/ITAM/2024/L003	LC	130	0.504	130	1	0.504	0.384	0.042
Apocynaceae	*Cryptolepis nigrescens *(Wennberg) L.Joubert & Bruyns	Globo	UHAS/ITAM/2024/L008	NE	127	0.492	127	1	0.492	0.378	0.040
Poaceae	*Andropogon gayanus* Kunth	-	UHAS/ITAM/2024/L009	NE	124	0.481	124	1	0.481	0.372	0.038
Musaceae	*Musa × sapientum* L.	Toworka	UHAS/ITAM/2023/L009	LC	121	0.469	121	1	0.469	0.366	0.037
Combretaceae	*Anogeissus leiocarpa* (DC.) Guill. & Perr.	Hehe/Hihe	UHAS/ITAM/2024/L004	LC	110	0.426	110	1	0.426	0.344	0.030
Rubiaceae	*Gardenia ternifolia* Schumach. & Thonn.	Efeti	UHAS/ITAM/2024/L005	LC	101	0.391	101	1	0.391	0.326	0.026
Fabaceae	*Leucaena leucocephala* (Lam.) de Wit	Klikagbe	UHAS/ITAM/2024/L006	NE	91	0.353	91	1	0.353	0.306	0.021
Arecaceae	*Eremospatha macrocarpa* Schaedtler	Mfia/cane	UHAS/ITAM/2024/L007	LC	83	0.322	83	1	0.322	0.290	0.017
Acanthaceae	*Avicennia germinans* (L.) L.	Amuti/ Amutsi	UHAS/ITAM/2024/L010	LC	81	0.314	81	1	0.314	0.286	0.016
Cyperaceae	*Cyperus papyrus* L.	Keti	UHAS/ITAM/2024/AP001	LC	79	0.306	79	1	0.306	0.282	0.016
Lamiaceae	*Tectona grandis* L.f.	Teak	UHAS/ITAM/2024/L011	EN	74	0.287	74	1	0.287	0.272	0.014
Arecaceae	*Cocos nucifera* L.	Neti	UHAS/ITAM/2021/FR008	NE	73	0.283	73	1	0.283	0.270	0.013
Fabaceae	*Tamarindus indica* L.	Eforti	UHAS/ITAM/2024/FR001	LC	68	0.264	68	1	0.264	0.260	0.012
Ebenaceae	*Diospyros mespiliformis* Hochst. ex A.DC.	Keyi/Keke	UHAS/ITAM/2024/L012	LC	67	0.260	67	1	0.260	0.258	0.011
Arecaceae	*Raphia hookeri* G. Mann & H. Wendl.	Alati	UHAS/ITAM/2024/L013	LC	62	0.240	62	1	0.240	0.249	0.010
Typhaceae	*Typha domingensis* Pers.	Ava	UHAS/ITAM/2024/L014	LC	61	0.236	61	1	0.236	0.247	0.009

UR = use report, CI = cultural importance, FC = frequency of citation, NU = number of uses, RFC = relative frequency of citation, RI = relative importance, CVe = cultural value

NE = not evaluated, LC = least concern, NT = Near Threatened, EN = endangered

Of the 26 species identified, the family Arecaceae (n = 6) was the most represented, followed by the Fabaceae (n = 4), Apocynaceae and Poaceae (n = 2; each) while the remaining 12 families were represented by a single plant species (n = 1; each). Ethnobotanical indices of the 26 species used by respondents in this study revealed the highest use report (UR) and cultural importance (CI) index for *Bambusa vulgaris* (n = 440, 1.705), followed by *Borassus aethiopum* (n = 406, 1.574), *Elaeis guineensis* (n = 397, 1.539), *Senna siamea* (n = 288, 1.116), *Azadirachta indica* (n = 265, 1.027), *Funtumia africana* (n = 199, 0.771), *Rhipsalis baccifera* (n = 187, 0.725), and *Raphia palma-pinus* (n = 175, 0.678) (Table 1). The number of uses (NU) for each plant species for the different building elements was found to be highest for *B*. *vulgaris* (n = 4), *S*. *siamea* and *A*. *indica* (n = 3; each), *B*. *aethiopum*, *E*. *guineensis*, *F*. *africana* (n = 2; each), while the remaining 20 species recorded a single NU (n = 1; each). The highest FC was recorded for *E*. *guineensis*, (n = 251), followed by *B*. *aethiopum* (n = 211), *R*. *baccifera* (n = 187), and *R*. *palma-pinus* (n = 175).

*E*. *guineensis* had the highest RFC value (0.973), followed by *B*. *aethiopum* (0.818), *R*. *baccifera* (0.725), and *R*. *palma-pinus* (0.678). The highest value for the relative importance index (RI) was recorded for *B*. *vulgaris* (0.823), an indication that it was the most frequently mentioned as useful and in the maximum number of uses (NU), followed by followed *E*. *guineensis* (0.750), *B*. *aethiopum* (0.670), *S*. *siamea* (0.588), *A*. *indica* (0.580), *F*. *africana* (0.525), *R*. *baccifera* (0.498) and *R*. *palma-pinus* (0.474). The cultural value index revealed that *B*. *vulgaris* was the most culturally valued with (CVe) value (0.714), followed by *E*. *guineensis* (0.499), *B*. *aethiopum* (0.429), *S*. *siamea* (0.231), and *A*. *indica* (0.205) (Table 1).

The agreement ratios among the respondents for the utilization of plant species for vernacular building elements were high (Table 2). The highest IAR was recorded for the wall lath (0.997) with only two plant species applied for this building element. Roof lath and binding material had the second highest IAR (0.994; each) followed by main beam (0.993), main pole and thatch material (0.991; each). The main poles construction element had the highest number of plant species (n = 12) followed by thatch material (n = 8), main beam (n = 6), roof laths and binding material (n = 4; each) and wall lath (n = 2). The S2 Table contains the IAR analysis of each plant species used to construct vernacular buildings.

**Table 2:** Number of use reports, number of species used, and informant agreement ratio (IAR) of plant species for the construction of particular vernacular building element in Ghana.

Building element	Nur	Ns	Nur-Ns	Nur-1	IAR
Main pole	1183	12	1171	1182	0.991
Main beam	730	6	724	729	0.993
Wall laths	339	2	337	338	0.997
Roof lath	539	4	535	538	0.994
Binding material	518	4	514	517	0.994
Thatch material	800	8	792	799	0.991

The specific valuable purposes determined by the fidelity level (FL) of the 26 plant species for vernacular building construction elements are presented in Table 3. Based on the FL, *B*. *vulgaris* was used purposefully for roof lath, while *E*. *guineensis* was for wall lath, and *B*. *aethiopum*, *F*. *africana*, and *S*. *siamea* were for the main beam. *A*. *indica B*. *ferruginea*, *D*. *mespiliformis*, *K*. *senegalensis*, *G*. *ternifolia*, *A*. *leiocarpa*, *T*. *indica*, *T*. *grandis*, and *B*. *ferruginea* were most useful for main poles. Pictures of the application of plant species for elements of vernacular buildings are provided in S1 File.

**Table 3:** Fidelity level (FL) of plant species used for vernacular building construction in Ghana.

Building element	Plants species used	FL (%)
Main poles	*Azadirachta indica*	100.00
	*Anogeissus leiocarpa*	100.00
	*Avicennia germinans*	100.00
	*Baphia nitida*	100.00
	*Gardenia ternifolia*	100.00
	*Diospyros mespiliformis*	100.00
	*Tamarindus indica*	100.00
	*Tectona grandis*	100.00
	*Bridelia ferruginea*	100.00
	*Antiaris toxicaria*	100.00
	*Senna siamea*	82.24
	*Funtumia africana*	44.20
Main beam	*Borassus aethiopum*	100.00
	*Funtumia africana*	100.00
	*Leucaena leucocephala*	100.00
	*Senna siamea*	100.00
	*Azadirachta indica*	70.87
	*Bambusa vulgaris*	67.90
Wall lath	*Elaeis guineensis*	100.00
	*Bambusa vulgaris*	54.32
Roof lath	*Bambusa vulgaris*	100.00
	*Borassus aethiopum*	92.42
	*Senna siamea*	86.92
	*Azadirachta indica*	86.41
Building rope	*Musa × sapientum*	100.00
	*Cryptolepis nigrescens*	100.00
	*Rhipsalis baccifera*	100.00
	*Eremospatha macrocarpa*	100.00
Thatch material	*Raphia hookeri*	100.00
	*Raphia palma-pinus*	100.00
	*Cyperus papyrus*	100.00
	*Cocos nucifera*	100.00
	*Typha domingensis*	100.00
	*Andropogon gayanus*	100.00
	*Elaeis guineensis*	58.17
	*Bambusa vulgaris*	49.38

## Discussion

Plant-based building materials are essential elements of vernacular buildings [9]. Nonetheless, not all plant species are considered valuable in vernacular construction by the local communities. Culturally important species typically have more than an average number of citations [30]. The high citations (use report) for *B*. *vulgaris*, *B*. *aethiopum*, *E*. *guineensis*, *S*. *siamea*, *A*. *indica*, and *F*. *africana* qualifies them as culturally significant species for vernacular building construction. Also, a UV of more than 1 indicates that the resource is highly valued in local communities and has a unique cultural significance [7, 31]. Thus, these plant species with a UV index exceeding 1 were highly valued as a source of plant-based material for vernacular buildings by construction artisans in local communities.

Bamboo, which was identified as the most highly valued and culturally important plant species for vernacular building construction, was consistently mentioned in other studies as applicable in vernacular building construction in Ghana [1, 32–34] and other places [2, 35, 36] although these studies did not calculate their use and cultural values. Agyekum et al. [1] found bamboo to be Ghana’s second most important vernacular building material after timber. The versatility of bamboo in construction elements in this study corroborates other studies that found it to be applied in various aspects of vernacular buildings [2, 35–37]. The high cultural importance of *B*. *aethiopum* for construction in this study is corroborated by other studies [38, 39] that also found it to be suitable for construction purposes.

The informant agreement ratio serves as a measure of the uniformity of ethnobotanical data [40]. The high IAR of plant species for vernacular building elements suggest that they have become essential to local cultural knowledge due to their effectiveness in vernacular housing construction. For instance, *A*. *leiocarpa*, *A*. *germinans*, *B*. *nitida*, *G*. *ternifolia*, *D*. *mespiliformis*, *T*. *indica*, *T*. *grandis*, *B*. *ferruginea*, and *A*. *toxicaria* were exclusively used for main poles, while *L*. *leucocephala* was used for the main beam only by all the respondents who mentioned these plant species. Likewise, all the respondents used *M*. *sapientum*, *C*. *nigrescens*, *R*. *baccifera*, and *E*. *macrocarpa* for fastening. At the same time, the respondents applied *R*. *hookeri*, *R*. *palma-pinus*, *C*. *papyrus*, *C*. *nucifera*, *T*. *domingensis*, and *A*. *gayanus* for thatching purposes only. According to Heinrich et al. [30], the selection and application of resources for traditional purposes are typically determined by how effective they are seen to be in the culture. The high IAR for the plant species for specific vernacular building elements indicates that they have not fallen into disuse from cultural adaption and are not perceived as ineffective for the purposes they are applied.

The Fidelity Level (FL) is useful for identifying the most preferred plant species used by vernacular building artisans to construct some aspects of the building. The purposes and species’ preferences for the construction elements identified in this study were consistent with their recorded application in other studies. Using bamboo for the main beam, wall, roof laths, and thatching material is well documented in other studies [2, 35–37]. Also, the use of *A*. *indica*, *B*. *nitida*, and *S*. *siamea* for house posts and rafters for vernacular houses is corroborated by previous studies [41–43]. The use of oil palm fronds and petiole for vernacular building elements in this study has been mentioned in previous studies [44–46]. The utilization of *T*. *domingensis*, *A*. *gayanus* and *C*. *papyrus* for thatch roof purposes is consistent with their documented application in other places [47–49]. *C*. *nigrescens* utilization for fastening purposes has been documented in Nigeria where the stem was used as a rope for tying domestic animals and firewood [50, 51]. However, the use of *R*. *baccifera* for tethering has yet to be reported in any previous study, which can be considered idiosyncratic to the study area. Peculiarities in ethnobotanical knowledge and applications in utilization have been identified in the study region relative to other areas of the country [7, 52].

## Conservation implications and valorization in sustainable construction

Rural communities with limited access to modern services and commodities rely heavily on exploiting natural resources for material purposes [7, 8]. Therefore, regardless of the rarity or conservation status of the resource or species, their use may persist, especially for species that have a high cultural importance. The harvest of culturally significant species for vernacular buildings in this study can be considered sustainable based on their conservation status. All the highly valued and culturally important species: *B*. *vulgaris*, *B*. *aethiopum*, *E*. *guineensis*, *S*. *siamea*, and *A*. *indica* are classified as Least Concern (LC) under the International Union for Conservation of Nature (IUCN) Red List Threatened Species [53], which have a lower risk of extinction.

Cultivated or domesticated plants that depend on humans for reproduction as well as species not harvested from vulnerable vegetation types and protected areas are less susceptible to overharvesting [11]. The culturally significant plant species in this study have been identified among the common plant species in the landscape [54]. Bamboo grows and matures faster than several other plant species widely distributed in Ghana, making their harvesting more sustainable for building construction [33, 34]. Oil palm plants are obtainable all year round and in considerable quantity, thus ensuring their abundance and availability [55]. *S*. *siamea* is one of Ghana’s most commonly planted species for afforestation [54]. The Neem tree is among Ghana’s most widely planted exotic plant species [54, 56]. The highly valued and culturally significant plant species in this study have the attributes of being abundant and widely distributed, with high reproductive and growth rates, cultivated to ensure that their harvest can be sustainably managed.

The sustainable valorization of resources is contingent upon the availability of species and their socio-economic value. The socio-economic value (i.e., ethnobotanical, economic, and nutritional values) and the species availability (i.e., their national distribution and threat status) form the basis for assigning valorization priority and plan, respectively [57]. All the highly valued and culturally significant species in this study have socio-economic value and species availability attributes for commercialization in Ghana. The valorization of highly valued and culturally important species can be an important resource for alleviating poverty in local communities. The study recommends exploring the culturally important plant species in green buildings in Ghana. Bamboo is established as a sustainable material for building construction [35, 37]. *B*. *aethiopum’s* potential for construction and its structural usefulness has been established [58]. Wahab et al. [55] identified the usefulness of oil palm fronds for board production as an alternative to timber wood. The environmental benefits and structural potentials of Neem trees for construction purposes have been studied [42, 56]. In the Ghanaian context, the importance of vernacular building materials for green construction is acknowledged [1, 32]. What is needed is the promotion of their adoption into the local building industry.

## Conclusion

Using a quantitative ethnobotanical approach, this study has established the plant species of high cultural significance for vernacular building construction elements in Ghana. The quantitative approach revealed the plant species’ actual cultural significance and value to local communities that previously had remained undetected. The study has established that bamboo is the most culturally significant and valuable species for vernacular building construction. The study found a high potential for valorization of the plant species with high cultural importance and value that the local government can promote for poverty alleviation. The culturally significant and valued species: *B*. *vulgaris*, *B*. *aethiopum*, *E*. *guineensis*, *S*. *siamea*, and *A*. *indica* for vernacular building construction are classified by the IUCN as Least Concern (LC), which have a lower risk of extinction. The study recommends vernacular building plant materials adoption for green construction in the formal construction industry and enhancement in the promotion of their valorization.

## Supporting information

S1 Table Data file used for analysis. (XLSX)

S2 Table Informant agreement ratio (IAR) analysis. (XLSX)

S1 File Pictures of vernacular building elements. (PDF)
