# Supplementary material for: Quantitative ethnobotany of plants used for vernacular building construction in Ghana
Source: PLoS One. 2024 Nov 15;19(11):e0313778. doi: 10.1371/journal.pone.0313778 (PMC11567639; doi:10.1371/journal.pone.0313778)
Supplement: S1 File — (PDF) [file pone.0313778.s003.pdf]

S1 File

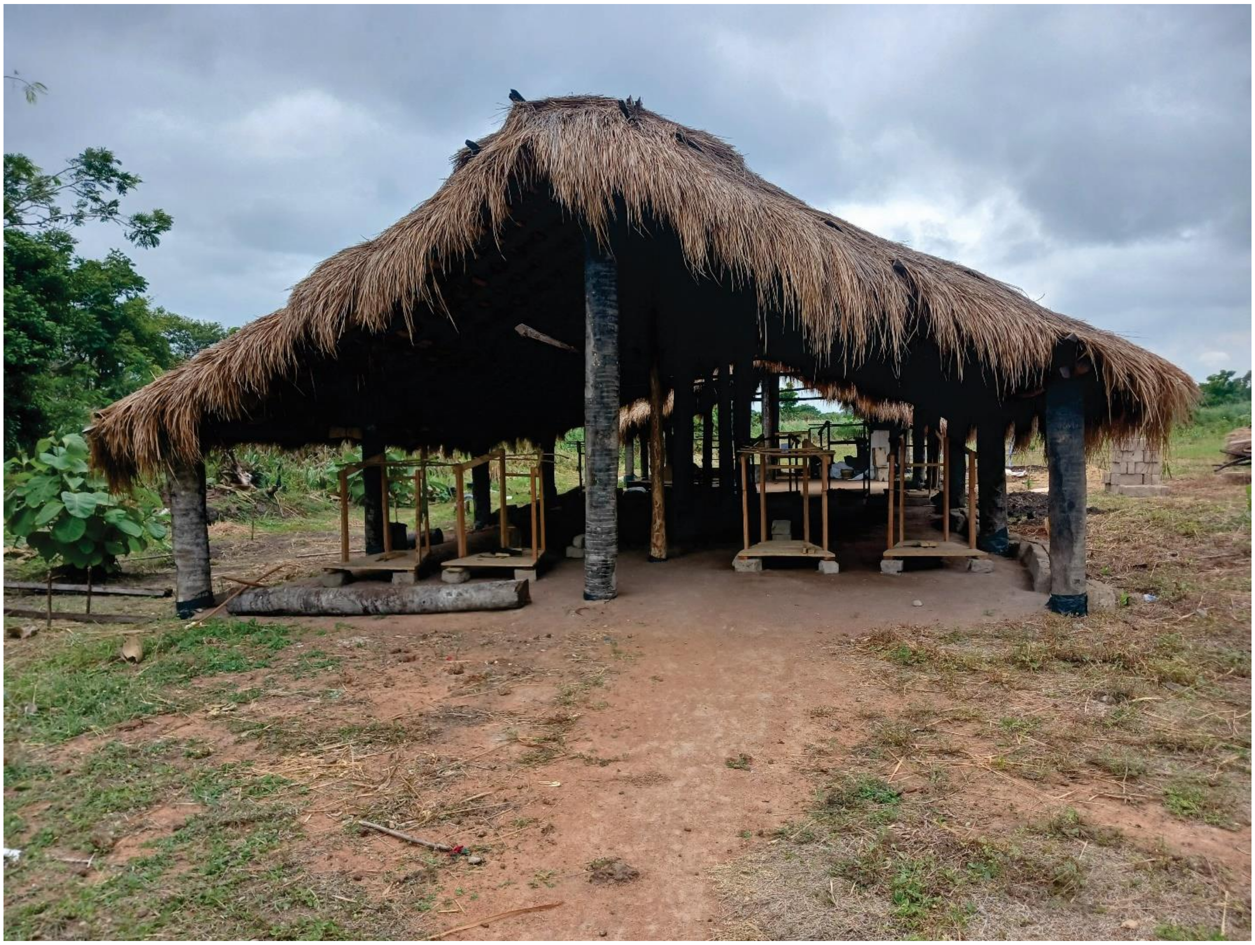

Vernacular building structure used as a community training center thatched with *Raphia palma-pinus* (Photo Credit: Maxwell Kwame Boakye, 2024)

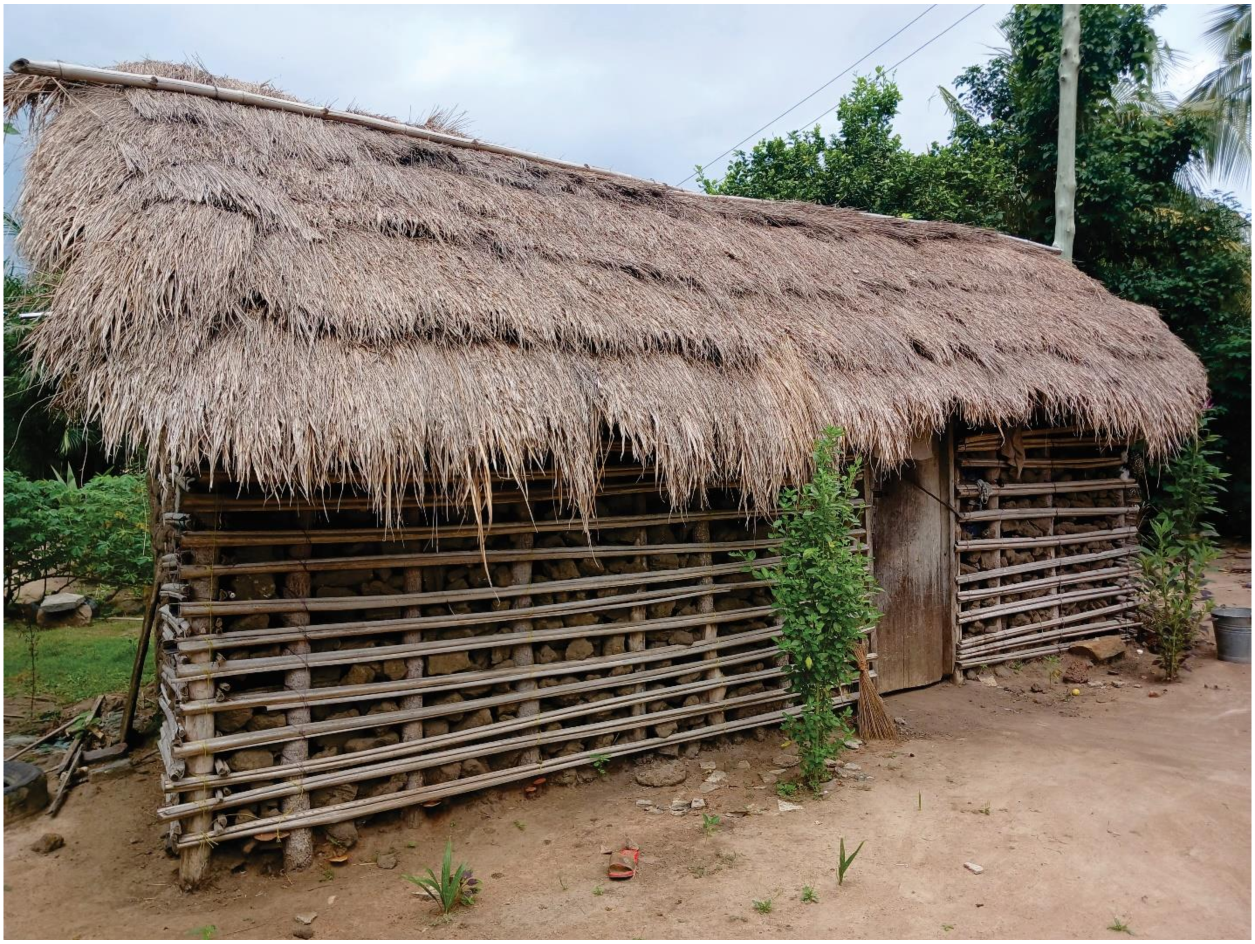

Vernacular building (Photo Credit: Maxwell Kwame Boakye, 2024)

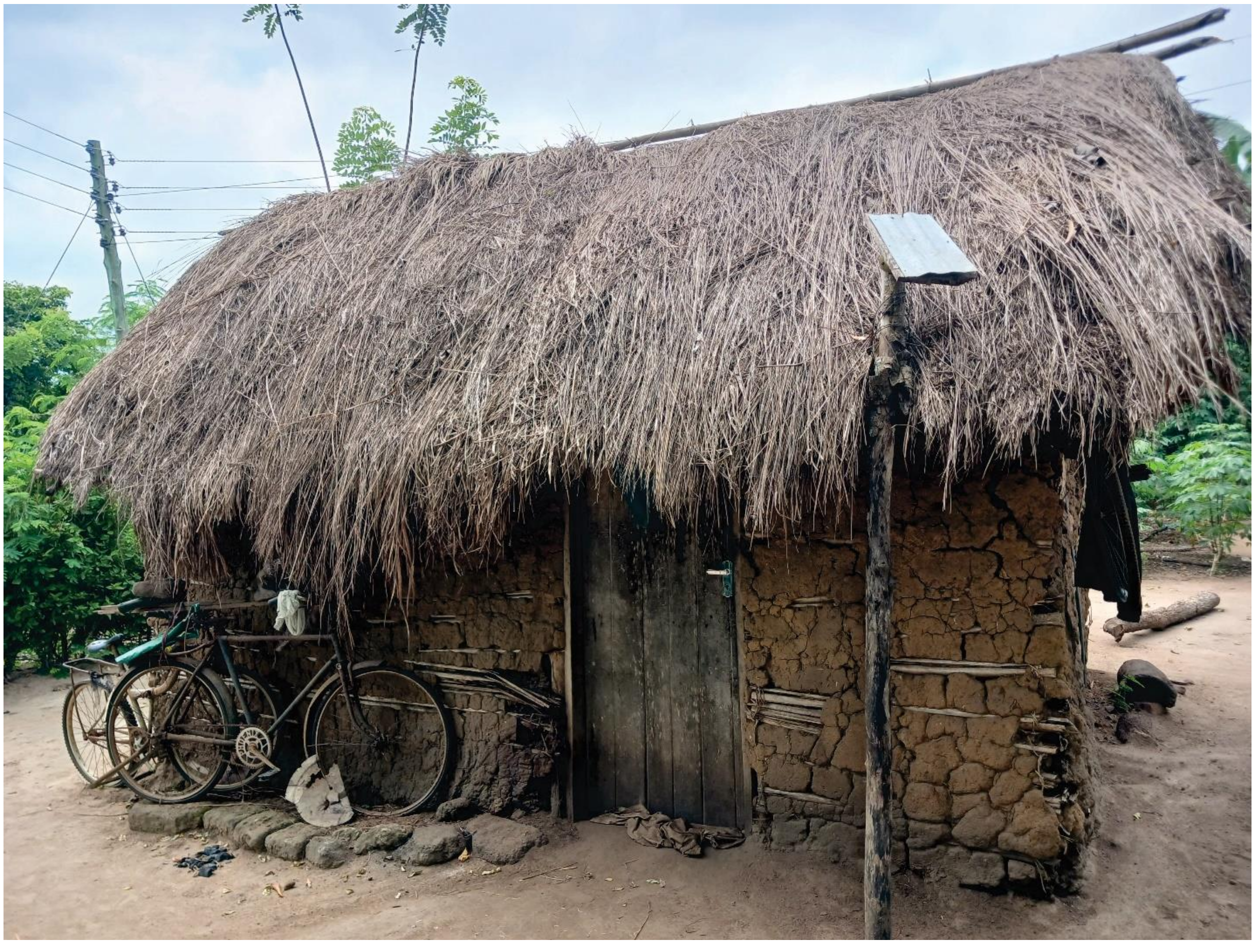

Vernacular building (Photo Credit: Maxwell Kwame Boakye, 2024)

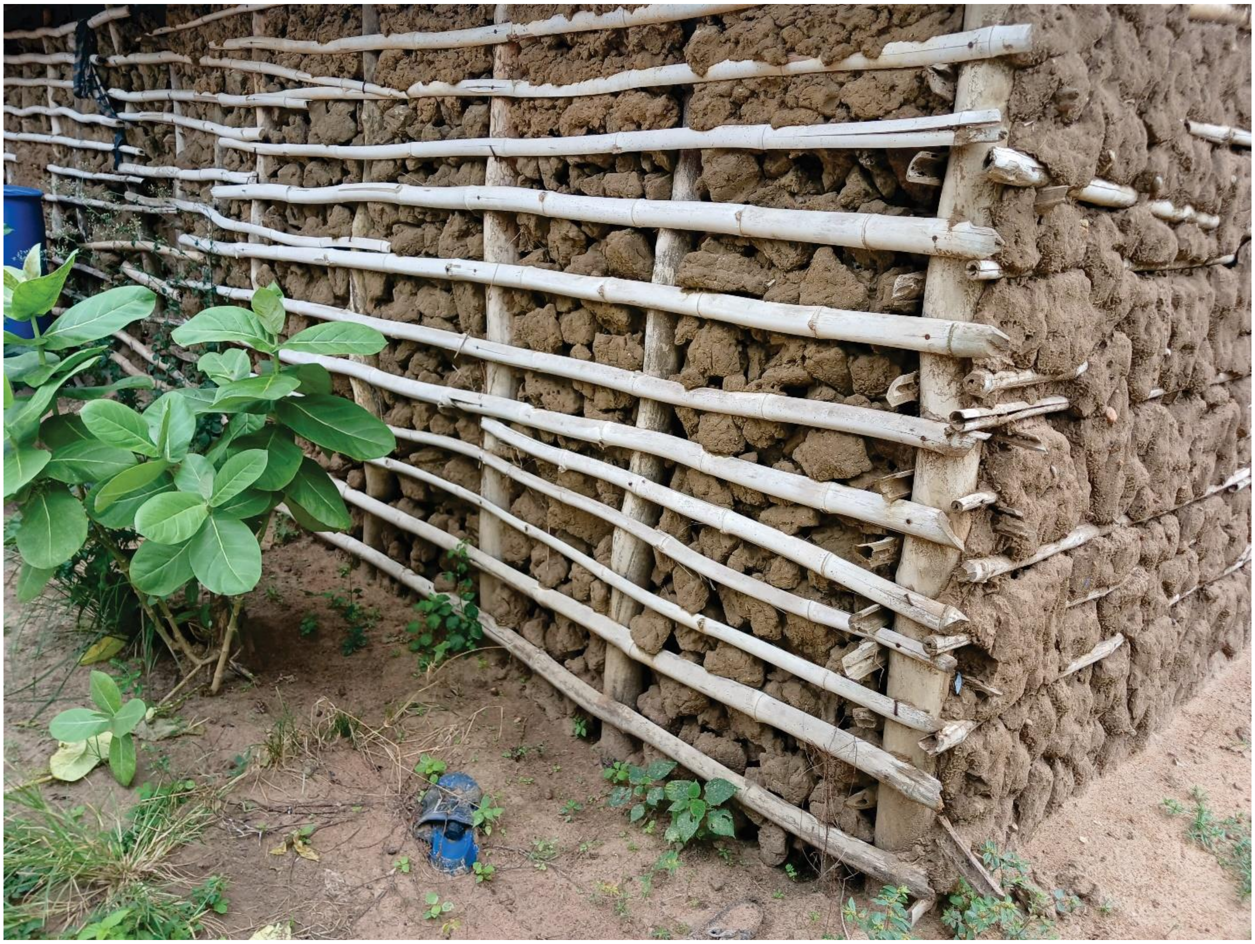

Bamboo wall lath of a vernacular building (Photo Credit: Maxwell Kwame Boakye, 2024)

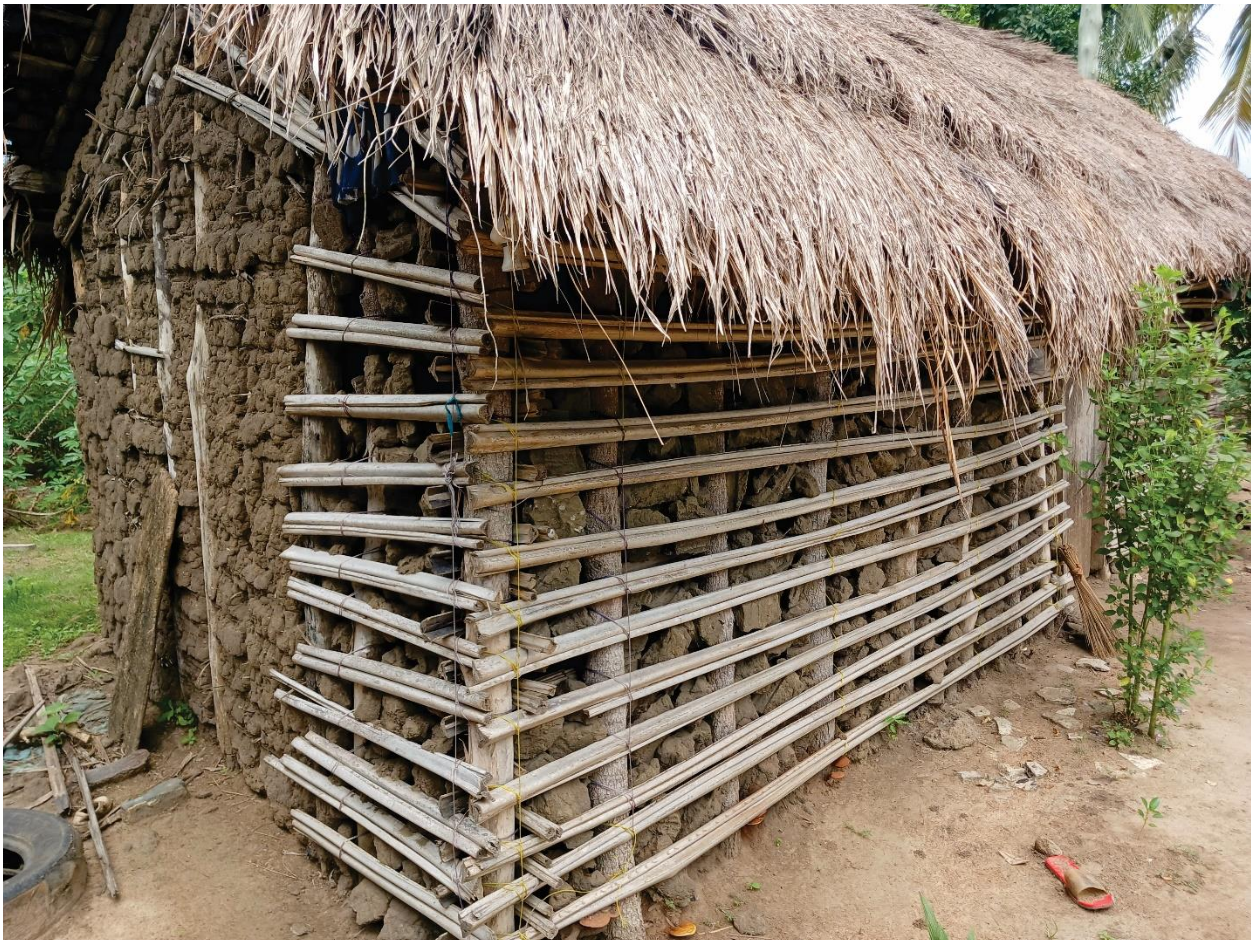

Elaeis guineensis petiole wall lath of a vernacular building (Photo Credit: Maxwell Kwame Boakye, 2024)

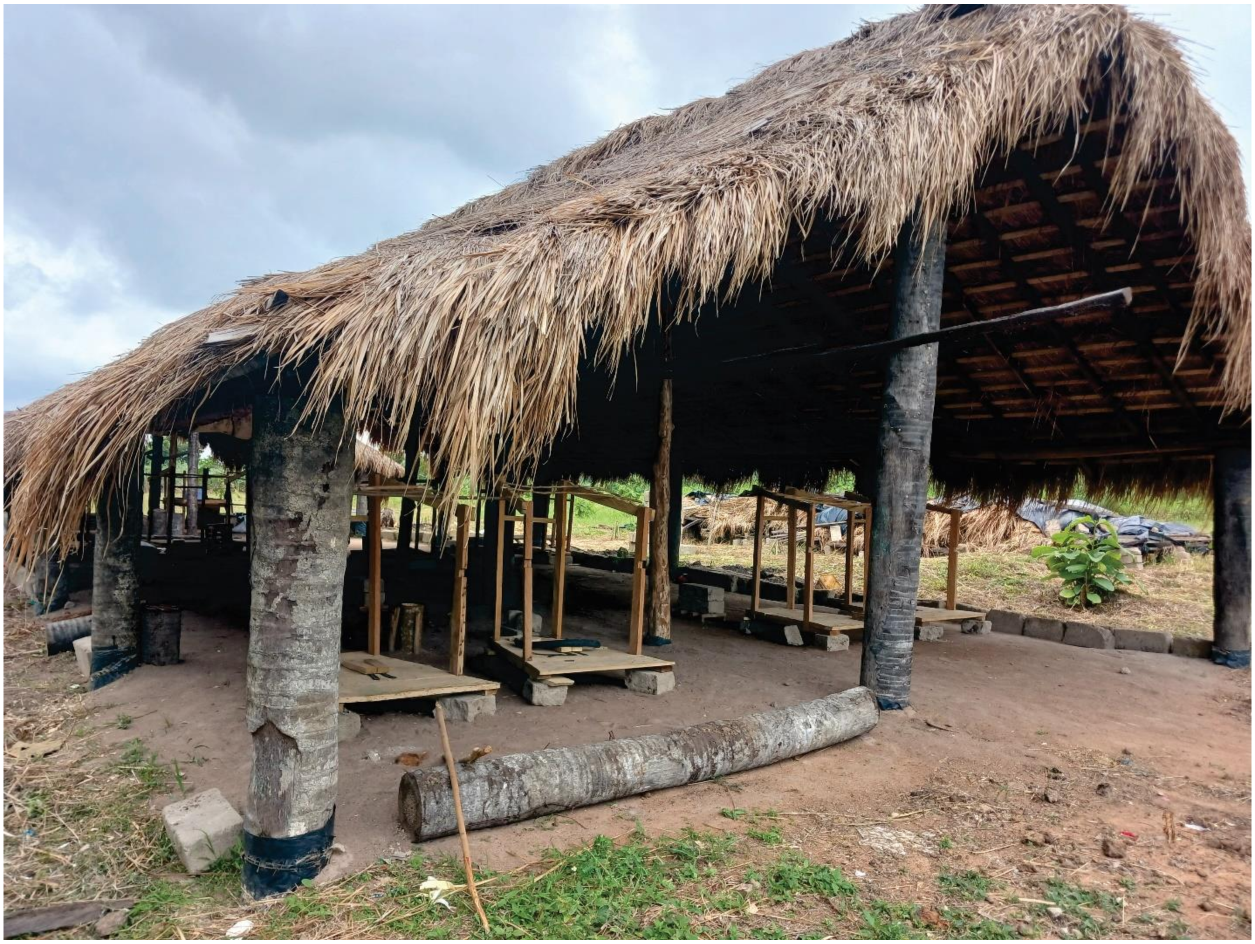

Borassus aethiopum used as the main poles (Photo Credit: Maxwell Kwame Boakye, 2024)

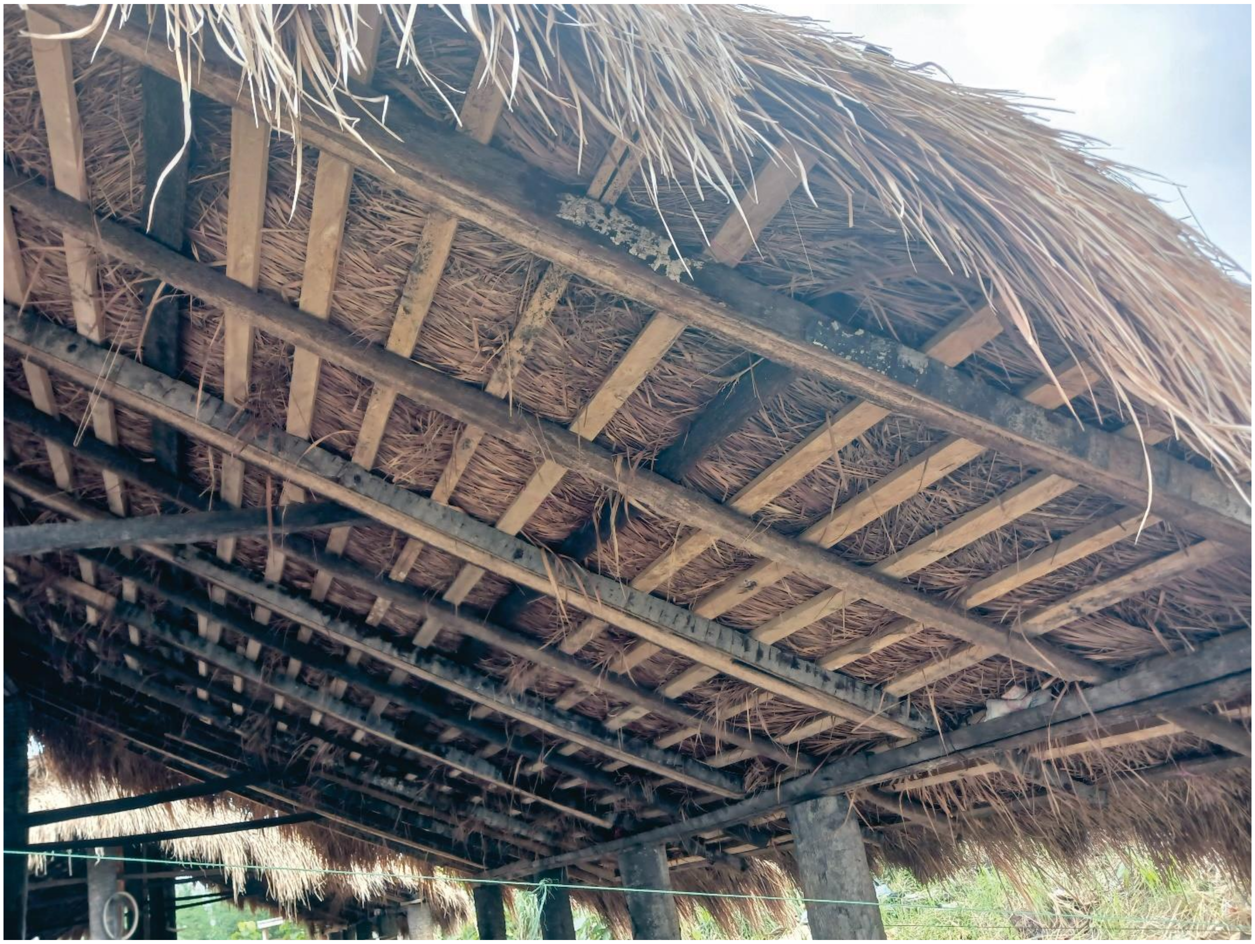

Sawn *Borassus aethiopum* used as roof laths (Photo Credit: Maxwell Kwame Boakye, 2024)

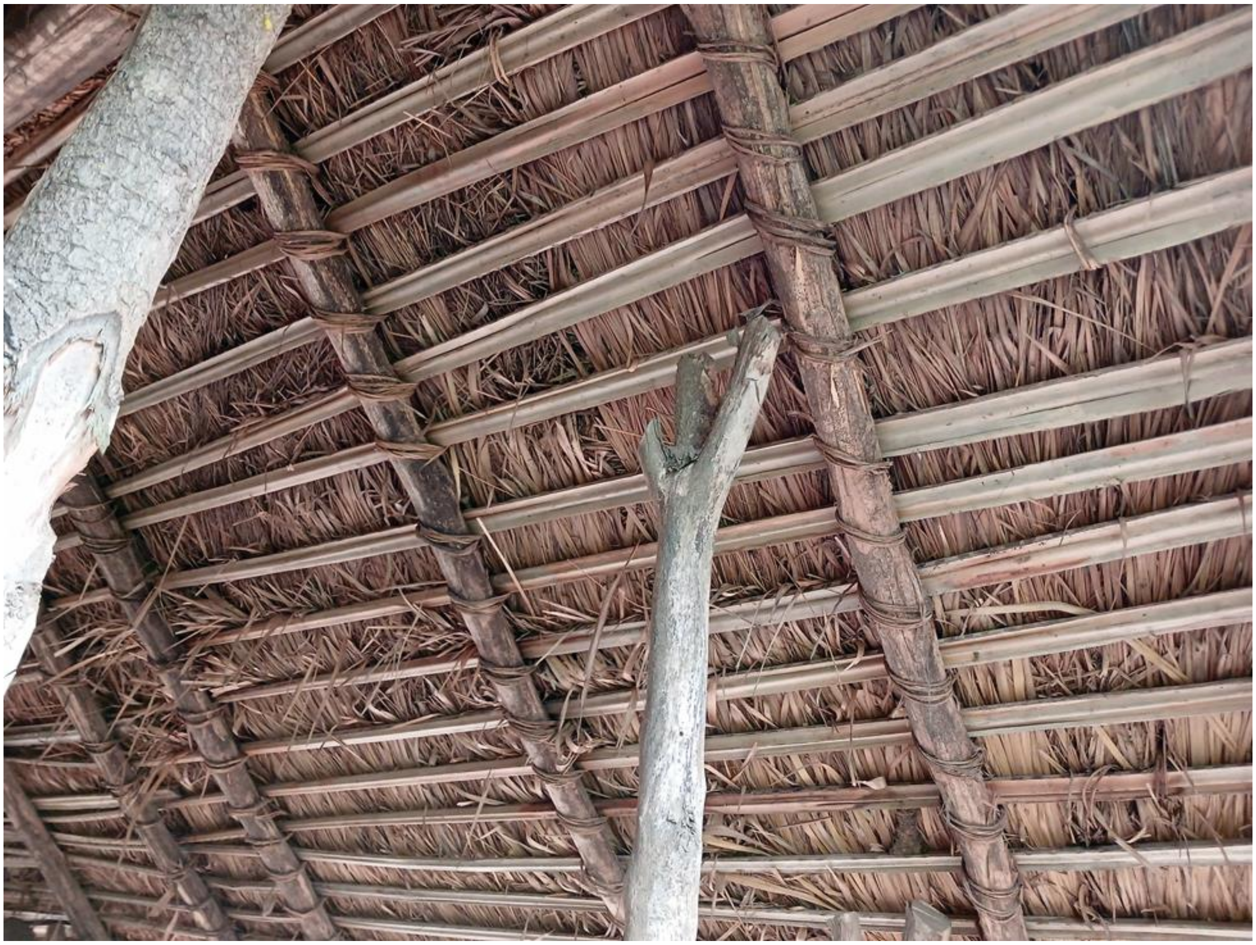

Elaeis guineensis petiole roof laths (Photo Credit: Maxwell Kwame Boakye, 2024)

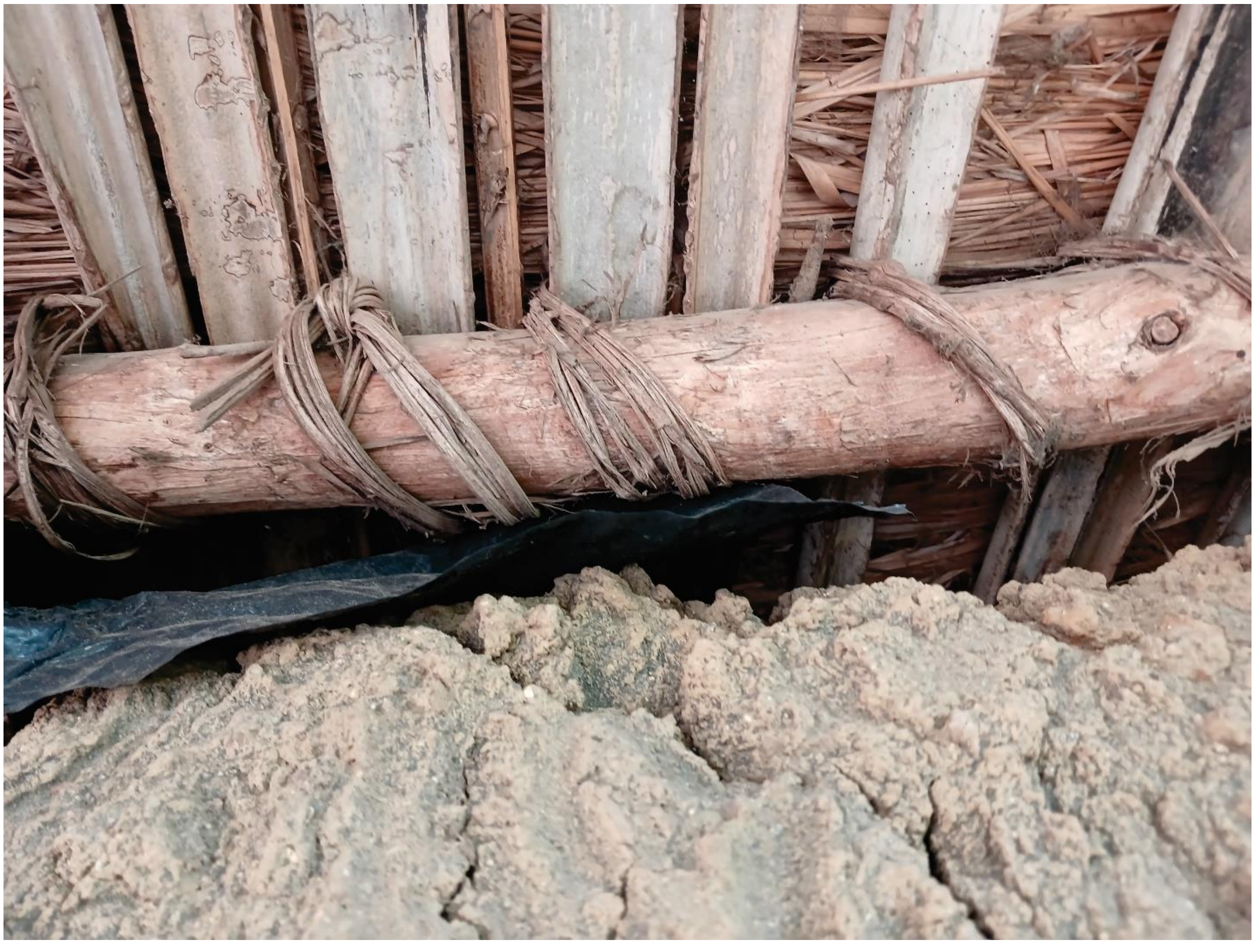

Tied laths with *Rhipsalis baccifera* (Photo Credit: Maxwell Kwame Boakye, 2024)
